# Sensitivity and Specificity of Common Autism Diagnostic Instruments for Early School-Aged Children

**DOI:** 10.3390/children13050680

**Published:** 2026-05-15

**Authors:** Maya J. Golden, Georgios Sideridis, Ellen Hanson, Stephanie J. Brewster, William Barbaresi, Elizabeth Harstad

**Affiliations:** 1 Division of Developmental Medicine, Boston Children’s Hospital, Boston, MA 02115, USA; maya.golden@childrens.harvard.edu (M.J.G.); georgios.sideridis@childrens.harvard.edu (G.S.); ellen.hanson@childrens.harvard.edu (E.H.); stephanie.brewster@childrens.harvard.edu (S.J.B.); william.barbaresi@childrens.harvard.edu (W.B.); 2 Rosamund Stone Zander and Hansjoerg Wyss Translational Neuroscience Center, Boston Children’s Hospital, Boston, MA 02445, USA

**Keywords:** autism spectrum disorder, ADOS-2, ADI-R, ROC curve, DSM-5

## Abstract

**Highlights:**

**What are the main findings?**

• In comparison to a best-estimate (BE) diagnosis made by a research psychologist, the Autism Diagnostic Observation Schedule, Second Edition (ADOS-2) and the Autism Diagnostic Interview-Revised (ADI-R) have good classification accuracies when used on their own, as well as when used in combination.

**What are the implications of the main findings?**

• Findings may inform decisions about which autism diagnostic measures to use, especially in cases where time and resources are limited.

**Abstract:**

**Background/Objectives**: This study assessed the diagnostic accuracy of two commonly used diagnostic instruments for autism spectrum disorder (ASD), the Autism Diagnostic Observation Schedule, Second Edition (ADOS-2) and the Autism Diagnostic Interview-Revised (ADI-R), in comparison to a best-estimate (BE) diagnosis made by a research psychologist. **Methods**: Two hundred and thirteen children aged 5 years 0 months to 7 years 11 months completed a comprehensive research assessment that included multiple diagnostic measures. Once each research assessment was complete, a research psychologist gave each participant an overall BE research diagnosis of Diagnostic and Statistical Manual of Mental Disorders, Fifth Edition (DSM-5) ASD based on all available information from diagnostic testing and behavioral observations during testing. We assessed sensitivity, specificity, positive predictive value (PPV), and negative predictive value (NPV) of both the ADOS-2 and ADI-R separately and in combination and used receiver operating characteristic (ROC) curves to compare the areas under the curve (AUCs) of these instruments. **Results**: Both the ADOS-2 Spectrum Criterion scoring (sensitivity = 96.2%; specificity = 97.5%) and ADOS-2 Autism Criterion scoring (sensitivity = 82.0%; specificity = 100%) had excellent accuracy in comparison to the BE ASD diagnosis. The ADI-R had good accuracy (sensitivity = 78.6%; specificity = 83.5%) compared to BE ASD diagnosis. In receiver operating curve analyses, both scoring criteria for ADOS-2 were significantly more accurate than the ADI-R. **Conclusions**: Overall, both instruments provide good, if not excellent, classification accuracies when used individually, as well as in combination. Thus, when deciding which measures to use for ASD research, other factors should also be considered.

## 1. Introduction

The diagnosis of autism spectrum disorder (ASD) is made clinically using the diagnostic criteria from the Diagnostic and Statistical Manual of Mental Disorders, Fifth Edition (DSM-5) [1]. A “best-estimate (BE) diagnosis”, conducted through observations of the child’s behaviors and collection of detailed information about the child’s developmental history and current functioning, is the recommended approach [2]. Diagnostic instruments were created to aid in the diagnostic process. For example, the Autism Diagnostic Observation Schedule, Second Edition (ADOS-2) [3] collects information about behavior through observation and directly administered tasks, and the Autism Diagnostic Interview-Revised (ADI-R) [4] provides information on current behaviors as well as developmental history from caregiver interviews. Both the ADOS-2 and ADI-R were developed before publication of the DSM-5, although they have been periodically updated to better reflect current ASD diagnostic criteria (as described below). Practical considerations, such as time and other resources, may preclude use of both the ADOS-2 and ADI-R. Thus, it is important to understand the accuracy of the ADOS-2 and ADI-R separately, and in combination, in relation to BE diagnoses for children with DSM-5 ASD.

**Figure 1:**
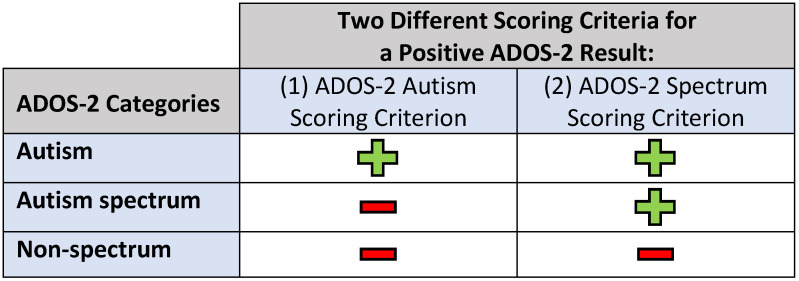
Two different scoring criteria for a positive ADOS-2 result. ADOS = Autism Diagnostic Observation Schedule. Diagnostic categories included in a positive score for each of the ADOS-2 scoring criteria are indicated by a “+”. Diagnostic categories included in a negative score for each of the ADOS-2 scoring criteria are indicated by a “−”.

The ADOS-2 is a semi-structured observation used to assess communication, social interaction, restricted and repetitive behavior, and play [3]. Administration of the ADOS-2 requires that the child have a nonverbal mental age of at least 12 months [5]. There are five different modules that can be used with children or adults with varying developmental and language levels. Each module differs slightly in its diagnostic algorithms and thresholds. Final summary scores on the ADOS-2 can fall within the categories of “autism”, “autism spectrum”, or “non-spectrum”. These three categorical results can be used to define a positive result on the ADOS-2 using two different scoring criteria: (1) ADOS-2 Autism Scoring Criterion differentiates between autism versus not autism (which includes the categories of autism spectrum and non-spectrum), and (2) ADOS-2 Spectrum Scoring Criterion differentiates between spectrum (which includes autism and autism spectrum categories) versus non-spectrum (Figure 1). A meta-analysis of 22 articles looking at the accuracy of the ADOS-2 in comparison to a BE diagnosis made after a comprehensive evaluation including both the ADOS-2 and a clinical interview showed that ADOS-2 sensitivities ranged from 0.89 to 0.92, and specificities ranged from 0.81 to 0.85 [6]. In a study assessing the different scoring criteria for the ADOS-2, the ADOS-2 Spectrum Scoring Criterion had higher sensitivities and lower specificities in comparison to the ADOS-2 Autism Scoring Criterion [2]. Also, the language level of the child may impact the correlation between ADOS-2 results and BE ASD diagnoses. Understanding how the ADOS-2 Spectrum and ADOS-2 Autism Criteria compare to other measures and how well the ADOS-2 works in children with varying language levels can influence future decisions about which ASD measures to use in research. Additionally, there is a lack of published work in which the research psychologist provided their report of whether or not the ADOS was “valid” in the context of their administration and consideration of the ADOS findings and determination of whether or not the child met BE ASD diagnosis.

The ADI-R is a semi-structured interview given to caregivers [4]. Questions in the interview fall into four categories: (1) reciprocal social interaction; (2) communication; (3) restrictive repetitive behavior; and (4) abnormality of development at or prior to 36 months. To administer the ADI-R to caregivers, their child must have a mental age above 24 months. In Western Psychological Services (WPS) scoring of the ADI-R, patients must meet or exceed defined cutoff scores in all four of the categories above to meet criteria for possible ASD [7]. The meta-analysis by Lebersfeld et al. [6] found that the mean sensitivity of the ADI-R was 0.75 and specificity was 0.82.

The ADOS-2 and ADI-R both require administration by trained personnel, are time-consuming, and may be costly to administer; thus, clinicians and researchers often use one diagnostic method or the other, rather than both [8]. Therefore, it is important to examine the accuracy of the ADOS-2 and ADI-R separately, and to compare that to the accuracy of using both the ADOS-2 and ADI-R results, in order to make informed decisions about how to best use these diagnostic instruments in research and clinical care.

The objectives of this study were to assess the accuracy of the ADOS-2 and ADI-R alone and in combination using standard detection signal measures (sensitivity, specificity, positive predictive value (PPV), negative predictive value (NPV), and receiver operating characteristic (ROC) curves for comparisons) in comparison to the reference standard BE ASD diagnosis. For the ADOS-2, different scoring criteria and differences in language modules were examined as well as a research psychologist report of validity for false negative and false positive cases. Based on the literature discussed above, we expect the ADOS-2 Spectrum Criterion to have a higher sensitivity and lower specificity compared to the ADOS-2 Autism Criterion. In addition, we predict that the ADOS-2 low language modules (modules 1 and 2) would have higher predictive validity compared to the ADOS-2 module 3 given that children with lower language levels may have more severe ASD symptom presentations, thereby facilitating clearer diagnostic classifications.

## 2. Materials and Methods

### 2.1. Recruitment and Study Participants

The present study uses data from a recently published prospective natural history study on the persistence of ASD in children diagnosed as toddlers [9]. Two hundred thirteen children aged 5 years 0 months to 7 years 11 months were recruited to complete a comprehensive research assessment. Recruitment occurred via mail, phone calls, or in-person recruitment when possible. All these children had previously received a DSM-5 ASD diagnosis between ages 12–36 months from a multidisciplinary clinical assessment at an outpatient developmental-behavioral pediatric clinic. Children who were non-English speaking, in custodial care, and/or with known genetic conditions associated with developmental outcomes (e.g., Down syndrome, Fragile X syndrome, PTEN mutation) were excluded. As previously published, when compared to a random subset of eligible nonparticipants from the same clinic, the study sample did not differ by age at diagnosis, cognitive ability, language, sex, race, or income. Participants did have higher adaptive and communication scores and fewer were of Hispanic ethnicity, compared with nonparticipants [9]. This study received ethical approval from the Boston Children’s Hospital IRB. Caregivers of participants provided written informed consent prior to participation.

### 2.2. Demographics

Parents provided demographic data including the child’s race/ethnicity, family income, and maternal education level through a questionnaire completed at the time of the research assessment.

### 2.3. Research Assessment at Age 5–7 Years

Research psychologists and supervised research assistants who were trained and research reliable in the administration of the ADOS-2 and ADI-R conducted the research assessments for this study from August 2018 to January 2022. Each participant’s research assessment included the ADI-R [4], ADOS-2 (module 1, 2, or 3) [3], Differential Abilities Scales-Second Edition (DAS-II) [10] or Bayley Scales of Infant and Toddler Development-Third Edition (Bayley-III) [11] for children whose cognitive abilities precluded administration of the DAS-II, Preschool Language Scales-Fifth Edition (PLS-5) [12], and Vineland Adaptive Behavior Scales-Third Edition Parent Interview (Vineland-3) [13]. Participants’ mental age was determined using the developmental/cognitive scores from the Bayley. Following the ADOS-2, the research psychologist responded “yes” or “no” to the question, “Was the ADOS-2 a valid representation of ASD symptoms?”. To document any situational factors that may have contributed to an invalid administration of the ADOS-2, the research psychologist was prompted to select one of the following reasons if their response about validity was “no”: noncompliance, hyperactivity, anxiety, or irritability. Once each research assessment was complete, a research psychologist with over 20 years’ experience assessing children with ASD gave each participant an overall BE research diagnosis of DSM-5 ASD (dichotomized to Persistent ASD or Non-Persistent ASD based on current functioning). This BE diagnosis was based on all available information from diagnostic testing and behavioral observations during testing. Given that the COVID-19 pandemic began in the middle of this study, 111 of the 213 research assessments were completed with facemasks. Although ADOS-2 administration and scoring are not validated using masks, the assessment procedures were performed as close to a standardized ADOS-2 administration as possible. For this study, we are not presenting data on research psychologist-reported validity of the ADOS for the 111 assessments completed with facemasks since the ADOS-2 has not yet been formally validated with the use of facemasks. Data on the impact of facemasks on ASD research assessments are presented in a separate manuscript [14].

### 2.4. Data Analyses

Methodology and approach to analyses for this manuscript were done in consideration of the STARD Checklist [15]. We used standard signal detection methods in the form of ROC curves and associated classification indices (i.e., sensitivity, specificity, PPVs, and NPVs) to report the accuracy of single and combined ADOS-2 and ADI-R algorithms in comparison to the reference standard BE ASD diagnosis [16].

**Figure 2:**
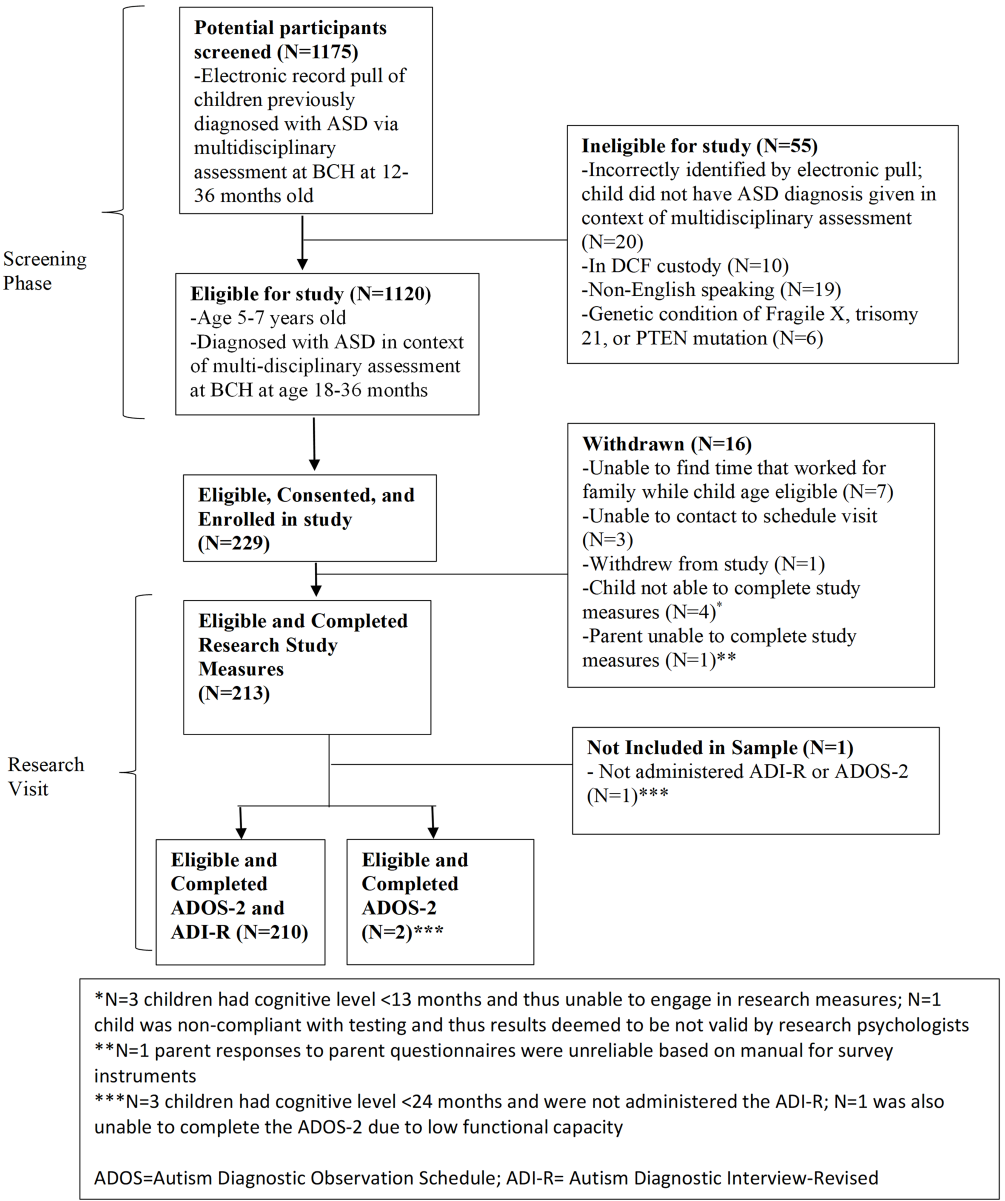
Flow diagram of potential participants.

The ADI-R was not administered to three of the 213 participants because their mental age was less than 24 months. One of these participants was also not administered the ADOS-2 due to low functional capacity. Therefore, a sample size of N = 210 was used for analysis of the ADI-R and combination of the ADOS-2 and ADI-R, while a sample size of N = 212 was used during analysis of the ADOS-2 alone (Figure 2). We assessed sensitivity, specificity, positive predictive value, and negative predictive value of the ADOS-2 and ADI-R separately and in combination using the following index test categories: (a) ADOS-2 Autism Scoring Criterion; (b) ADOS-2 Spectrum Scoring Criterion; (c) ADOS-2 Spectrum Scoring Criterion comparing children with low language modules (1 and 2) versus those with ADOS-2 module 3; (d) ADI-R using WPS scoring; (e) meeting both ADOS-2 Spectrum Scoring Criterion and ADI-R (most stringent condition); and (f) meeting either ADOS-2 Spectrum Scoring Criterion or ADI-R (least restrictive condition).

To assess research psychologist-reported validity of the ADOS-2, we identified ADOS-2 false positives (those who met ADOS-2 cutoffs for autism or autism spectrum but did not receive a BE ASD diagnosis) and false negatives (those who did not meet ADOS-2 cutoffs for autism or autism spectrum but received a BE ASD diagnosis) and looked at the psychologist-reported scores of validity for those participants. These analyses were done for the 102 subjects seen prior to the pandemic (without facemasks).

ROC analyses were used to evaluate each algorithm’s ability to correctly predict a BE ASD diagnosis by comparing the sensitivity and specificity calculated for each of the conditions described above. The areas under the curve (AUCs) for the various conditions were estimated using the Hanley and McNeil [17] methodology, and significant differences were determined using a two-tailed test and a nominal α level of 0.05. Power for the ROCs was estimated using a large sample variance approximation [17]. Given that the predictor variable of BE ASD diagnosis had 131 participants with ASD and 79 participants without ASD, power of 80% was achieved with an AUC of 0.61 at an alpha level of 5%. Thus, this study was adequately powered to detect small-to-moderate ROC effects.

Power for contrasting two ROCs was conducted using a difference of 0.1 (e.g., between 0.80 and 0.90). A sample size of n = 206 is associated with power equal to 80% for an alpha level of 5% (two-tailed test) when contrasting the area between two AUCs that differ by 0.10.

## 3. Results

In this study of 213 children, the mean age of participants at the time of the research assessment was 74.3 months (SD = 7.1). Thirty-six (16.9%) of the participants were female, while the other 177 (83.1%) participants were male. The majority (80.3%) of children were White, and 69.9% had private insurance (Table 1). Of the 212 participants who were administered the ADOS-2, 41 (19.3%) received module 1, 27 (12.7%) received module 2, and 144 (67.9%) received module 3.

### 3.1. Sensitivities, Specificities, and Positive and Negative Predictive Values for ADOS-2 and ADI-R

As shown in Table 2, the ADOS-2 Spectrum Scoring Criterion had a high predictive validity of the BE ASD diagnosis: sensitivity (96.2%), specificity (97.5%), PPV (98.5%), NPV (93.9%). The ADOS-2 Autism Scoring Criterion had sensitivity of 82.0% and specificity of 100%. All 68 children who were administered the low language ADOS-2 modules (modules 1 or 2) had ADOS-2 Spectrum Scoring results that perfectly matched their BE ASD diagnoses (giving these ADOS-2 modules 100% sensitivity and specificity). In comparison, ADOS-2 module 3 had a lower sensitivity (92.6%) and specificity (97.4%). Sensitivity and specificity of the ADI-R were 78.6% and 83.5%, respectively.

In assessing combinations of the ADOS-2 and ADI-R, the least restrictive condition, which required a child to meet the cutoff for ASD on either the ADOS-2 Spectrum Scoring Criterion or the ADI-R, had the highest possible sensitivity (100%) and lowest specificity across all conditions (81.0%). Conversely, the most stringent condition, where a child had to meet the ASD cutoff on both the ADOS-2 Spectrum Scoring Criterion and ADI-R, had the lowest sensitivity (74.8%) and the highest possible specificity (100%).

### 3.2. Research Psychologist-Report of ADOS-2 Validity

Using the ADOS-2 Spectrum Scoring Criterion in relation to the BE ASD diagnosis, there were no false positive cases and two false negatives (Table 3). For the two false negative cases, the research psychologist reported that the ADOS-2 was a valid representation of ASD symptoms. In contrast, the ADOS-2 Autism Scoring Criterion yielded no false positives and 13 false negatives. Again, the research psychologist reported that the ADOS-2 was a valid representation of ASD symptoms for all of these 13 cases.

### 3.3. ROC Analyses and Comparisons Between Diagnostic Instruments

According to Gallop et al. [18], 90–99% AUC is considered “excellent”, 80–89% is “good”, 70–79% is “fair”, and 60–69% is “poor”. The ADOS-2 Spectrum Scoring Criterion modules 1 and 2 had 100% classification accuracy. The following had “excellent” classification accuracies (Table 4): the ADOS-2 Spectrum Scoring Criterion for all modules together, ADOS-2 Autism Scoring Criterion, ADOS-2 module 3, and a combination of using either the ADOS-2 Spectrum Scoring Criterion or ADI-R. The ADI-R alone and the combination of both the ADOS-2 Spectrum Scoring Criterion and ADI-R had “good” classification accuracies.

**Figure 3:**
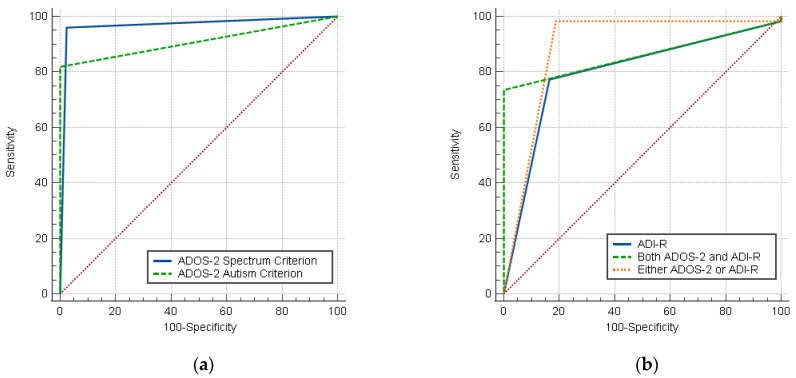
(**a**) Receiver operator characteristic (ROC) curves for ADOS-2 Spectrum and Autism Criteria (N = 212). (**b**) Receiver operator characteristic (ROC) curves for ADI-R and combined ADOS-2 Spectrum Criterion and ADI-R (N = 210). ADOS = Autism Diagnostic Observation Schedule; ADI-R = Autism Diagnostic Interview-Revised.

As measured by the AUC, the predictive validity of the ADOS-2 Spectrum Scoring Criterion (0.969) was significantly greater than the predictive validity of the ADOS-2 Autism Scoring Criterion (0.910), *D* = 0.059, *p* = 0.019 (Figure 3a). Additionally, the ADOS-2 Spectrum Scoring Criterion had significantly higher predictive validity compared to the ADI-R (0.811), *D* = 0.158, *p* < 0.001. The lower language level modules (1 and 2) of the ADOS-2 Spectrum Scoring Criterion (1.000) had significantly higher predictive validity compared to module 3 of the ADOS-2 (0.950), *D* = 0.050, *p* = 0.017.

## 4. Discussion

The present study used data from research assessments of 213 children ages 5–7 years old to investigate the diagnostic accuracy of the ADOS-2 and ADI-R (separately and combined) in comparison to the reference standard BE DSM-5 ASD diagnosis. Our findings are broadly in agreement with past research on the sensitivity and specificity of the ADOS-2 and ADI-R [6]. All of the index test algorithms (ADOS-2 Autism Scoring Criterion, ADOS-2 Spectrum Scoring Criterion, ADOS-2 low language (1 + 2) modules, ADOS-2 module 3, ADI-R, either ADOS-2 Spectrum Scoring Criterion or ADI-R, both ADOS-2 Spectrum Scoring Criterion and ADI-R) had “good”, if not “excellent”, classification accuracies [18]. However, there were statistically significant differences between the classification accuracies of the algorithms, which are discussed below and may be considered to inform decisions about which ASD diagnostic instruments to use in research assessments.

Similar to the findings of Risi et al. [2], our results demonstrate that expanding the ADOS-2 Scoring Criterion from Autism to Spectrum increased sensitivity while maintaining a high specificity. The ADOS-2 Spectrum Scoring Criterion was able to correctly identify 19 of the 24 cases that had been missed (false negatives) when the Autism Scoring Criterion was applied. This is not surprising given that the ADOS-2 Spectrum Scoring Criterion more closely aligns with the DSM-5 ASD criteria which was used in making the BE ASD diagnosis in this study. Furthermore, the ROC analyses highlight that the ADOS-2 Spectrum Scoring Criterion had significantly higher predictive validity compared to the ADOS-2 Autism Scoring Criterion. Despite this significant difference, however, both algorithms had “excellent” classification accuracy overall [18].

To our knowledge, this study is the first that collected the research psychologist’s report of validity for ADOS-2 scores of participants who had discrepancies between their ADOS-2 assessment results and BE ASD diagnoses. In all discrepant cases (for both the ADOS-2 Autism and ADOS-2 Spectrum Criteria), the administering research psychologist reported that the ADOS-2 was a valid representation of ASD symptoms. Therefore, while the ADOS-2 sometimes provided a different assessment result than the BE diagnosis, the difference was not because the ADOS-2 scores were poor representations of the participants, but rather that there might be additional necessary information obtained for a BE ASD diagnosis that is not covered by the ADOS-2 alone.

We also considered the impact of participant language level on the classification accuracy of the ADOS-2. The ADOS-2 low language modules (modules 1 and 2) and ADOS-2 module 3 had “excellent” classification accuracies, but the predictive validity of ADOS-2 modules 1 and 2 was significantly higher. The decision about which ADOS-2 module to administer is guided by the child’s language level. Thus, children administered modules 1 or 2 had lower language levels than those administered module 3. Given that language challenges are a fundamental component of ASD, it is possible that the ADOS-2 might be more accurate in classifying children with more severe ASD presentations. This is consistent with De Bildt et al. [19] who found that the probability of agreement between the ADOS classification and the DSM-IV autism diagnosis increased with increased autism symptoms in a sample of 128 children and adolescents with intellectual disability.

The sensitivity and specificity of the ADOS-2 Spectrum Scoring Criterion were higher than that of the ADI-R. ROC analyses indicated that the ADOS-2 Spectrum Scoring Criterion had “excellent” classification accuracy, and the ADI-R had “good” classification accuracy [18]. A previous meta-analysis and systematic review reported that the ADOS-2 was more accurate than the ADI-R, and thus the ADOS-2 should be considered for any ASD assessment [6]. However, our analyses show that while the ADOS-2 classification accuracy was significantly higher, both algorithms provide “good” if not “excellent” classification accuracies individually, as well as when used in combination in the research setting.

While the current paper focuses on the diagnostic accuracy of the ADOS-2 and ADI-R, it is important to consider that these diagnostic instruments obtain their data from different sources as the ADOS-2 utilizes behavioral observations of the child while the ADI-R utilizes the parent/caregiver report of past developmental history and current behaviors. In studies assessing the correlation between the ADOS-2 and ADI-R when used in children, the correlations have been reported to be between 0.51 and 0.71 [2,20,21]. The ADOS-2 reflects the child’s current behavioral functioning while the ADI-R includes the parent report of current and previous social communication skills and behaviors. Given that the ADOS-2 and ADI-R both have at least “good” classification accuracy, factors such as priorities regarding information source, administration length, staff training, and other measures used should also help inform which diagnostic instrument(s) to use in an ASD assessment.

There are some limitations to consider when interpreting the findings of this study. All the children in this study were recruited from a single multidisciplinary developmental clinic at an academic medical center. While the sample was broadly representative of the population of patients seen in the clinic, most children were White and had educated, affluent mothers. In order to determine the generalizability of these results, additional research is needed across a range of other settings. Additionally, unlike other previously published validation studies conducted in a diagnostically naive sample, all participants in the present study received ASD diagnoses prior to their school-age research visits. Although this may have resulted in higher calculated predictive validities than the published literature, recent research describing how ASD diagnoses can change across individuals’ lifetimes highlights the need to identify measures that can be used to assess for ASD at various moments in time [9]. Another limitation to acknowledge is that during research assessments, the clinician making the BE ASD diagnosis was not blind to the results of the ADOS-2 and ADI-R and often conducted one or both assessments. The order in which the ADOS-2 and ADI-R were administered was not standardized across participants, but the BE diagnosis was always made after both the ADOS-2 and ADI-R were complete. It is possible that the clinician’s BE ASD diagnoses were biased by the clinician’s awareness of the ADOS-2 and ADI-R scoring. However, these findings nonetheless add to the growing literature on the use of these measures in research settings. This bias may have resulted in an overestimation of predictive validities compared to other publications. There is an inherent tension in that clinical BE ASD diagnoses typically rely on the use of such standardized instruments. Thus, to be consistent with clinical standards of practice, the BE ASD diagnoses made in this research setting needed to factor in behavioral observations from the ADOS-2 and parent report from the ADI-R. Future work could examine the predictive validity of the ADOS-2 and ADI-R in comparison to BE ASD diagnoses that were made by a trained clinician who was blind to the scores of the two instruments. The present study analyzed subjective, non-validated information from the research psychologist about whether or not they thought the ADOS-2 was a valid assessment. However, it is important to note that this study was not designed to collect inter-rater reliability at the time of assessment given that research protocols utilizing the ADOS-2 and ADI-R typically require reliability to be established prior to research administration. An additional limitation to the present study is that the COVID-19 pandemic began mid-way through the study and approximately half (111/213) of the research assessments were conducted with facemasks given hospital requirements (notably, for this study, analyses of research psychologist-reported validity of ADOS-2 did not include the subjects seen during the pandemic given the validity concerns). The present study did not collect data on whether the 111 children assessed during the COVID-19 pandemic wore masks for the full ADOS-2, part of the ADOS-2, or only during other parts of the research visit, which limits our ability to control for facemask use in our analyses. However, our team previously published that the use of facemasks had minimal to no impact on the overall assessment results in this sample [14]. All the above-mentioned factors may have impacted the results of this study. Despite this, this study’s findings reflect the administration of the ADOS-2 and ADI-R within an ASD research study by a research psychologist who has over 20 years of experience conducting ASD research assessments. The research psychologist followed all existing protocols and expert guidance related to administration of these diagnostic measures.

The findings of this study suggest that in the research setting, both the ADOS-2 and the ADI-R have good classification accuracies in comparison to the BE ASD diagnosis. The ADOS-2 newer scoring criterion, specifically ADOS-2 Spectrum Scoring Criterion, outperformed the ADOS-2 Autism Scoring Criterion. Although both performed well, the ADOS-2 Spectrum Scoring Criterion had higher sensitivity and specificity than the ADI-R. When deciding which measures to use for ASD assessment in research, multiple factors (including accuracy, decisions about data from behavioral observations or parent report, time and resource restrictions, and psychologist/clinician availability) should be considered. The findings of this study may inform decisions about which measures to use for research, especially in cases where resources are limited.

## Figures and Tables

**Table 1:** Demographic information for total sample.

Demographic and Sociodemographic Data	Total Sample N (%) (N = 213)
Age at Research Assessment (months)	
Mean (SD)	74.27 (7.1)
Module of ADOS-2 Administered (During Research Assessment)	(N = 212) ^a^
Module 1	41 (19.3%)
Module 2	27 (12.7%)
Module 3	144 (67.9%)
Sex	
Female	36 (16.9%)
Male	177 (83.1%)
Race ^b^	
American Indian/Alaskan	1 (0.5%)
Asian	14 (6.6%)
Black/African American	19 (8.9%)
Hawaiian/Pacific Islander	0 (0.0%)
White	171 (80.3%)
Other ^c^	18 (8.4%)
Missing/Prefer not to answer	3 (1.4%)
Ethnicity	
Hispanic/Latino	30 (14.1%)
Not Hispanic/Latino	171 (80.3%)
Prefer not to answer	1 (0.5%)
Missing	11 (5.2%)
Insurance Type	
Private	149 (69.9%)
Public	49 (23.0%)
Tri-Care	11 (5.2%)
Self-pay	0 (0.0%)
Other or Unknown ^d^	4 (1.9%)
Maternal Education Level	
Did not attend high school	0 (0.0%)
Some high school	1 (0.4%)
High school graduate/GED	17 (8.0%)
Trade or vocational school	3 (1.4%)
Associate’s degree	18 (8.5%)
Some college	29 (13.6%)
Bachelor’s Degree	68 (31.9%)
Graduate/Professional Degree	77 (36.2%)
Prefer not to answer/Missing	0 (0.0%)

Categories for race, ethnicity, and insurance were defined based on the demographic categories used in the electronic health record system. Participants were counted as “Missing” if they did not provide a response for the variable. ^a^ One of the 213 participants was not administered the ADOS due to their low functional capacity at the time of testing. ^b^ N = 12 reported ≥1 race. These categories were provided to the parent who then marked which of the categories best described their child. ^c^ Parents selected “other” for race if they felt that none of the other provided options applied to their child. ^d^ Unknown and missing grouped together on our survey.

**Table 2:** Sensitivities, specificities, and positive and negative predictive values of separate and combined ADOS-2 and ADI-R.

Index	Sensitivity % (95% CI)	Specificity % (95% CI)	PPV % (95% CI)	NPV % (95% CI)
**ADOS-2 (Autism)** (N = 212) ^b^	82.0 (74.6–87.6)	100 (95.4–100)	100 (96.6–100)	76.7 (67.3–84.5)
**ADOS-2 (Spectrum)** (N = 212) ^b^	96.2 (91.5–98.4)	97.5 (91.3–99.3)	98.5 (94.6–99.6)	93.9 (86.5–97.4)
**ADOS-2 Modules 1 and 2** (N = 68) ^c^	100 (94.5–100)	100 (29.2–100)	100 (94.5–100)	100 (29.2–100)
**ADOS-2 Module 3** (N = 144) ^c^	92.6 (83.9–96.8)	97.4 (90.9–99.3)	96.9 (89.5–99.2)	93.7 (86.0–97.3)
**ADI-R** (N = 210) ^a^	78.6 (70.6–85.3)	83.5 (73.5–90.9)	88.8 (81.6–93.9)	70.2 (59.9–79.2)
**Both ADOS-2 (Spectrum) and ADI-R** (N = 210) ^a^	74.8 (66.5–82.0)	100 (95.4–100)	100 (96.3–100)	70.5 (61.2–78.8)
**Either ADOS-2 (Spectrum) or ADI-R** (N = 210) ^a^	100 (97.2–100)	81.0 (70.6–89.0)	89.7 (83.6–94.1)	100 (94.4–100)

Note: Sample sizes for each of the ROC analyses are noted above. ^a^ Three of the 213 participants were not administered the ADI-R because their mental ages were less than 24 months. ^b^ One of these participants was also not administered the ADOS. ^c^ Of the 212 participants administered the ADOS, 41 were administered module 1, 27 were administered module 2, and 144 were administered module 3. PPV = positive predictive value; NPV = negative predictive value; ADOS = Autism Diagnostic Observation Schedule; ADI-R = Autism Diagnostic Interview-Revised.

**Table 3:** Validity of discrepant ADOS-2 scores.

Instrument	Number	Was ADOS Valid?
ADOS-2 Autism Scoring Criterion False Positives	0	Valid: 0 Not Valid: 0
ADOS-2 Autism Scoring Criterion False Negatives	13	Valid: 13 Not Valid: 0
ADOS-2 Spectrum Scoring Criterion False Positives	0	Valid: 0 Not Valid: 0
ADOS-2 Spectrum Scoring Criterion False Negatives	2	Valid: 2 Not Valid: 0

N = 102. ADOS = Autism Diagnostic Observation Schedule.

**Table 4:** Receiver operator characteristic (ROC) analyses for separate and combined ADOS-2 and ADI-R.

Index	AUC	AUC 95%	SE	z-Test	AUC *p*-Value
**ADOS-2 (Autism) ^a^** (N = 212)	0.910	0.863–0.945	0.021	19.807	<0.001 ***
**ADOS-2 (Spectrum) ^a^** (N = 212)	0.969	0.935–0.988	0.014	33.466	<0.001 ***
**ADOS-2 Modules 1 and 2 ^b^** (N = 68)	1.000	0.947–1.000	0.000	-	-
**ADOS-2 Module 3 ^b^** (N = 144)	0.950	0.901–0.979	0.021	21.110	<0.001 ***
**ADI-R (WPS)** (N = 210)	0.811	0.751–0.861	0.032	9.749	<0.001 ***
**Both ADOS-2 (Spectrum) ** **and ADI-R** (N = 210)	0.874	0.821–0.916	0.024	15.394	<0.001 ***
**Either ADOS-2 (Spectrum) ** **or ADI-R** (N = 210)	0.905	0.857–0.941	0.027	15.172	<0.001 ***

Note: Three participants were not administered the ADI-R because their mental ages were less than 24 months. One of these participants was also not administered the ADOS. Sample sizes for each of the ROC analyses are noted above. *** *p* < 0.001. ^a^ The difference between the AUCs for the ADOS-2 ASD criterion and ADOS-2 autism criterion was significant, D = 0.059, *p* = 0.019. ^b^ The difference between the AUCs for the ADOS-2 low modules (1 and 2) and the ADOS-2 module 3 was significant, D = 0.050, *p* = 0.017. ADOS = Autism Diagnostic Observation Schedule; ADI-R = Autism Diagnostic Interview-Revised; WPS = Western Psychological Services scoring; AUC = area under the curve.

## Data Availability

This dataset is not publicly available due to privacy restrictions. The raw data supporting the conclusions of this article will be made available by the authors on request.

## Abbreviations

The following abbreviations are used in this manuscript:

ADI-R	Autism Diagnostic Interview-Revised
ADOS-2	Autism Diagnostic Observation Schedule, Second Edition
ASD	Autism spectrum disorder
AUC	Area under the curve
Bayley-III	Bayley Scales of Infant and Toddler Development-Third Edition
BE	Best estimate
DAS-II	Differential Abilities Scales-Second Edition
DSM-5	Diagnostic and Statistical Manual of Mental Disorders, Fifth Edition
IRB	Institutional review board
NPV	Negative predictive value
PLS-5	Preschool Language Scales-Fifth Edition
PPV	Positive predictive value
ROC	Receiver operating characteristic
Vineland-3	Vineland Adaptive Behavior Scales-Third Edition Parent Interview
WPS	Western Psychological Services
